# Application of Internet of Things in Cell-Based Therapy Delivery: Protocol for a Systematic Review

**DOI:** 10.2196/16935

**Published:** 2020-03-31

**Authors:** Ching Lam, Michelle Helena van Velthoven, Edward Meinert

**Affiliations:** 1 Digitally Enabled PrevenTative Health Research Group Department of Paediatrics University of Oxford Oxford United Kingdom; 2 Department of Primary Care and Public Health Imperial College London London United Kingdom

**Keywords:** internet of things, IoT, childhood obesity, wearables, physical activity tracking

## Abstract

**Background:**

Internet of Things (IoT), or Industry 4.0, represents a smart shift to more interconnected manufacturing processes where individual entities within the supply chain communicate with each other to achieve greater flexibility and responsiveness in general manufacturing and leaner manufacturing to reduce the cost of production. IoT has become instrumental in driving leaner manufacturing and more efficient systems in other industries such as transportation and logistics. Cell-based therapeutic products could potentially transform various diseases; however, the delivery of these products is complex and challenging.

**Objective:**

This study aims to understand the applicability of IoT in cell-based product supply chains and delivery.

**Methods:**

We will search Medline, EMBASE (OvidSP), Web of Science, Cochrane Library &amp; HEED, Scopus, ACM digital library, INSPEC, ScienceDirect, and the IEEE Xplore Digital Library for studies published after 2008 using a combination of keywords and subject headings related to IoT used in cell therapies. Additionally, a Google search to identify gray literature will be conducted. Two authors will independently screen the titles and abstracts identified from the search and accept or reject the studies according to the study inclusion criteria. Any discrepancies will then be discussed and resolved. The quality of the selected literature will be assessed using the Critical Appraisal Skills Programme systematic review checklist.

**Results:**

Data from eligible publications will be abstracted into a predesigned form to map the current and future directions of the technologies, applications, benefits, and challenges in the implementation of IoT in regenerative medicine. This study will be published in a peer-reviewed journal in accordance with the Preferred Reporting Items for Systematic Reviews and Meta-Analyses guidelines. This systematic review will be executed by June 2020, and the completed review will be published in a peer-reviewed journal to inform future developments in IoT application for the delivery of cell-based therapies.

**Conclusions:**

This review paper will provide an overview of all technologies available in the area and inspect the current IoT applications in cell-based therapies to identify the benefits, challenges, and future directions of using IoT to allow safe and cost-effective delivery of cell-based therapies.

**International Registered Report Identifier (IRRID):**

PRR1-10.2196/16935

## Introduction

### Background

Internet of Things (IoT) is a concept first proposed by Ashton [1] at a presentation at Procter & Gamble (Cincinnati, Ohio) in 1999. By collecting data from individual things and using these data efficiently to facilitate the exchange of information within the network, material and product flows can be tracked to achieve more accurate and real time visibility of the supply chain, which improves communication between entities and streamlines the different steps in the supply chain.

Over the last two decades, IoT has been successfully implemented in various industries including aerospace, aviation, automotive, telecommunications, medicine and health care, independent living, pharmaceutical, retail, logistics and supply chains, manufacturing, process, environmental, transportation, agricultural and breeding, media and entertainment, recycling, and insurance [2,3]. Various benefits have been realized through the application of IoT. Constant monitoring and real-time data acquisition and analysis lead to benefits such as earlier detection of risks, faster response times, and cost savings. This has a wide range of usages from mine production safety [4] to home health care applications [5]. Usage of radio frequency identification (RFID), sensor technologies, and networking architecture, allows better perception and transmission of underground environment information, which leads to better and more timely decision making and safer mine production. Miniature wearable sensors are used to monitor older adults and patients with chronic conditions in order to allow monitoring beyond the boundaries of hospitals. This improves the standard of health care reducing labor costs by lowering the required amount of contact hours with medical staff. Interoperability from connecting information from one domain to others allows better use of resources [6]. One of the most significant applications is in smart highway systems where sensors and cameras are used to collect traffic information that then feeds into a real-time update of an indicator’s operating rules on the highway such as speed limit [7].

A survey of the trends and topics in IoT research conducted by Whitmore et al [8] in 2015 looked into the evidence base available in the IoT research space and identified gaps in literature for specific applications and governance frameworks for the regulation of IoT. The implementation of IoT is generally divided into three layers—the sensing layer, the architecture layer, and the application layer [9]. In the context of cell therapies, which are therapies where whole living cells are administered to patients for treatment of diseases, Harrison et al [10] reviewed the application of fluorescent optical sensors on cell therapy manufacturing, and connecting real-time metabolic measurements to quality attributes of products, which could allow more scalable processes and better quality products. Outside of manufacturing, sensors can also be used for in vivo monitoring postadministration [11].

Technology platforms for facilitating information flow and exchange have been made available by industry and academic partnerships. Vitruvian Networks, for instance, is a partnership set up by GE Ventures (Menlo Park, California) and Mayo Clinic (Rochester, Minnesota) to make use of biomarkers, cell therapy processes, and clinical outcome data to guide therapy development [12]. IoT allows real-time control, more integrated maintenance, better adaptability to changing demands, better collaboration across the supply chain of cell therapies, better traceability, smarter products, and new business models for continuous improvement of products [13]. Frameworks for distributed manufacturing and service-oriented IoT deployments have been proposed and discussed in previous literature [9,14-17], but for highly regulated manufacturing environments such as for cell-based therapies, the interface between IoT architecture and Good Automated Manufacturing Practice [18] has not been documented. To allow successful implementation of IoT, the challenges specific to the area have to be identified. Issues such as privacy and security encountered in other IoT applications [19] have to be identified and considered in a manner specific to cell therapy manufacturing and delivery to inform a tailored IoT approach that facilitates translation and delivery of cell-based products.

This paper aims to systematically review all technologies available in the area, and inspect the current IoT application in cell-based therapies to identify the benefits, challenges, and future directions of using IoT to allow safe and cost-effective delivery of cell-based therapies.

### Aims and Objectives

The aim of this review is to understand the current available technologies for IoT, the extent of implementation in cell-based therapy delivery, and the drivers and challenges for implementation of IoT. This will allow the gaps for IoT implementation to be identified and will inform future directions in cell therapy supply chain optimization.

### Key Research Questions

Our research questions are as follows:

1. What is the current state of IoT implementation in cell-based therapy delivery? What companies and partnerships are engaged in IoT (especially for autologous therapies)?
2. What are the technologies available for IoT specific to cell-based therapy delivery?
3. What are the benefits of IoT in cell-based therapy delivery?
4. What are the challenges of implementing IoT on cell-based therapy delivery?

## Methods

### Study Design

This systematic review will be conducted following the PRISMA (Preferred Reporting Items for Systematic Reviews and Meta-Analyses) guidelines (Multimedia Appendix 1) [20].

In accordance with the PRISMA-P (Protocols) checklist recommendation, the inclusion criteria of this protocol are in accordance with the PICO (Participant, Intervention, Comparator, and Outcomes) standards. Details of the PICO to be included in the review are described in Textbox 1.

**Participant, Intervention, Comparator, and Outcomes and study type review inclusion criteria.**

**Participant**

- Patients receiving cell-based therapy

**Intervention**

- Any supply chain step using or interacting with the Internet of Things (IoT) technologies/networks in a cell-based therapy delivery process, from patient cell collection (if autologous) or cell sourcing (if allogeneic) to postadministration monitoring and follow-up

**Comparator**

- No intervention, standard practice, or any other type of intervention that is not IoT such as paper-based batch records

**Outcomes**

- Benefits and challenges of IoT implementation in tissue or cell-based therapy delivery

**Study type**

- Any study type (study type will not be subjected to any restrictions)
- Publications between 2008-2018
- English publications

### Exclusion Criteria

The fields of both IoT and cell therapy are rapidly advancing; therefore, to ensure that studies found are up to date, only publications from 2008-2018 will be considered. Studies not published in the English language are excluded due to a language barrier. Studies relevant only to the discovery, research, and development phases of cell-based therapies are not in the scope of this review.

### Search Strategy

The following databases will be searched: Medline, EMBASE (OvidSP), Web of Science, Cochrane Library & HEED, Scopus, ACM digital library, INSPEC, ScienceDirect, and the IEEE Xplore Digital Library. In addition, a search on Google to identify gray literature will be conducted. Table 1 shows the search concepts and keywords to be searched for this review. Search strings will be constructed using cell-based therapy keywords and keywords from other concepts. A sample search was conducted on the PubMed database, and the search strings used are described in Table 2.

**Table 1:** Search terms.

Concept	Keywords
Cell-based therapeutics	Cell* therapy
IoT^a^	Industry 4.0, smart factory, business intelligence, internet of things, IoT
IoT technologies	Sensor, biosensor, monitoring device, machine learning, barcode, RFID^b^, Auto ID, WSN^c^, Cloud computing, paperless
Communication	remote operation, automated workflow, data-driven, traceability, (network AND automat*)
Data security	Privacy, security, GAMP^d^, data integrity
Data management	Data stor*, data analy*

^a^IoT: Internet of Things.

^b^RFID: radio frequency identification.

^c^WSN: wireless sensor network.

^d^GAMP: Good Automated Manufacturing Practice.

**Table 2:** Sample search conducted on the PubMed database.

Search string	Number of results
(“cell* therapy”) AND (IoT OR Industry 4.0 OR ”business intelligence“ OR ”internet of things“)	42
(”cell* therapy”) AND (sensor OR biosensor OR “monitoring device” OR “machine learning” OR “barcode” OR “RFID” OR “Auto ID” OR “WSN” OR “Cloud computing” OR “paperless”)	70
(“cell* therapy”) AND (“remote operation” OR “automated workflow” OR “data-driven” OR “traceability” OR (“network” AND automat*))	21
(”cell* therapy”) AND (Privacy OR security OR GAMP OR data integrity)	41
(“cell* therapy”) AND (”data stor*“ OR (data analy*))	74
Total results from PubMed search	248
Duplicates removed	170

### Study Selection

EndNote X8 software will be used for the removal of duplicates. Textbox 1 describes the inclusion criteria of the review. Two independent reviewers will screen the titles and abstracts of papers to ensure no bias occurs. Papers that are ineligible will be eliminated, and the full text of those that appear to meet the review’s eligibility criteria will be obtained and read in full to ensure eligibility. Any contradictions or discrepancies between the reviewers that arise will be discussed until consensus is reached.

### Quality Assessment and Risk of Bias

Two reviewers will independently check each article to minimize bias using the collaboration’s risk of bias tool as described in the Cochrane Handbook for Systematic Review of Interventions [21]. All selected articles will be judged for their quality based on the Critical Appraisal Skills Programme systematic review checklist [22] and data analysis.

### Data Extraction

The eligible literature will then be described for its three-layer architecture of IoT proposed in various literature [9,23]. Any observed benefits and challenges of IoT in cell therapy delivery will be extracted and mapped against its status of implementation identified in the included studies. A sample data abstraction form can be found in Textbox 2. The data extracted will then be analyzed qualitatively.

**Sample data abstraction form.**

**Current practice/company and future directions for the items below**

**Sensing layer:**

Sensing devices and technologies such as radio frequency identification tags

**Network layer:**

Service entities arrangement, virtual entity and information, resource modules

**Application layer:**

Functionalities of Internet of Things

**Observed benefits (if any)**

**Observed challenges (if any)**

## Results

The study aims to provide a landscape of the application of IoT technologies in the cell therapy field and identify the gaps and challenges for IoT application. A sample search was conducted on the PubMed database, and the search strings used are described in Table 2. This systematic review will be executed by June 2020, and the completed review will be published in a peer-reviewed journal to inform future developments in IoT application for the delivery of cell-based therapies.

## Discussion

This review paper will provide an overview of all technologies available in the area and inspect the current IoT application in cell-based therapies to identify the benefits, challenges, and future directions of using IoT to allow safe and cost-effective delivery of cell-based therapies.

## Appendix

Multimedia Appendix 1 Preferred Reporting Items for Systematic review and Meta-Analysis Protocols (PRISMA-P) 2015 Checklist.

## Abbreviations

IoT: Internet of Things
P: protocol
PICO: Participant, Intervention, Comparator, Outcomes
PRISMA: Preferred Reporting Items for Systematic Reviews and Meta-Analyses
RFID: radio frequency identification
